# Predictors of Cardiac Sparing in Deep Inspiration Breath-Hold for Patients With Left Sided Breast Cancer

**DOI:** 10.3389/fonc.2018.00564

**Published:** 2018-11-27

**Authors:** Abbas Mkanna, Osama Mohamad, Paul Ramia, Ranim Thebian, Maha Makki, Hani Tamim, Wassim Jalbout, Bassem Youssef, Toufic Eid, Fady Geara, Bilal Shahine, Youssef H. Zeidan

**Affiliations:** ^1^Department of Radiation Oncology, American University of Beirut, Beirut, Lebanon; ^2^Department of Radiation Oncology, University of Texas Southwestern Medical Center, Dallas, TX, United States; ^3^Biostatistics Unit, Clinical Research Institute, American University of Beirut Medical Center, Beirut, Lebanon

**Keywords:** breast cancer, radiotherapy, breath-hold, DIBH, smoking

## Abstract

**Purpose:** The purpose of this study was to evaluate patient-related non-dosimetric predictors of cardiac sparing with the use of deep inspiration breath-hold (DIBH) in patients with left-sided breast cancer undergoing irradiation (RT).

**Materials and Methods:** We retrospectively reviewed charts and treatment plans of one-hundred and three patients with left-sided breast cancer. All patients had both free-breathing (FB) and DIBH (with body surface tracking) plans available. (MHD) and V4 (heart volume receiving at least 4 Gy) were extracted from dose volume histograms. Fisher's exact and Chi-square tests were used to identify predictors of reductions in MHD and V4 after DIBH.

**Results:** One-hundred and three patients were identified and most underwent mastectomy. MHD and V4 decreased significantly in DIBH plans (0.74 ± 0.25 Gy vs. 1.72 ± 0.98 Gy, *p* < 0.0001 for MHD; 4 ± 4.98 cc vs. 20.79 ± 18.2 cc, *p* < 0.0001 for V4). Body mass index (BMI), smoking and timing of CT simulation (spring/winter vs. summer/fall) were significant predictors of reduction in MHD whereas BMI, field size, chemotherapy, axillary dissection, and timing of CT simulation predicted reduction in V4. On multivariate analysis, BMI, and timing of CT simulation remained significant predictors of the heart-sparing effect of DIBH.

**Conclusions:** In the setting of limited resources, identifying patients who will benefit the most from DIBH is extremely important. Prior studies have identified multiple dosimetric predictors of cardiac sparing and hereby we identified new non-dosimetric factors such as BMI and timing of treatments.

## Introduction

Radiotherapy (RT) reduces recurrences and improves cancer-specific survival for patients with breast cancer (1–3). However, RT is associated with secondary exposure of the heart and other vital organs to “collateral” radiation injury. A well-cited investigation revealed that the risk of major coronary events increases linearly by 7.4% for every 1 Gy increase in mean heart dose (MHD) (4). The long-term risk of cardiac morbidity and mortality after irradiation (RT) for left-sided breast cancer is controversial especially for patients undergoing treatment in the current era (4–6). Modern RT techniques have significantly reduced cardiac exposure (7) but the long-term effects of modern RT on cardiac disease remain unknown.

Deep inspiration breath-hold (DIBH) increases the physical separation between the heart and chest wall and thus reduces cardiac exposure during intact breast or chest wall RT (8, 9). Not all patients, however, benefit equally from DIBH. Several studies investigated predictors of cardiac benefit from DIBH with several candidate measures such as maximal heart distance, and cardiac contact distance (10–13). Developing patient selection algorithms for DIBH is essential to optimize workflow thus eliminating the need for CT simulation and/or treatment plans in both the free-breathing (FB) and DIBH conditions for all patients with left-sided breast cancer especially in the setting of limited resources (machines, time, etc.). In this study, we aimed to evaluate patient-related non-dosimetric predictors of cardiac sparing with the use of DIBH in patients with left-sided breast cancer undergoing irradiation.

## Materials and methods

### Patient population

This study was from a single institution and was approved by the institutional review board. Between December 2015 and May 2017, patients with left-sided breast cancer who underwent whole breast or chest wall irradiation after lumpectomy or mastectomy were identified. Data on patient characteristics such as age, body mass index, and smoking status, in addition to medical co-morbidities [congestive heart failure (CHF), prior chest surgery, prior lung disease, or presence of simultaneous psychiatric conditions] were collected. Treatment factors such as tumor stage, type of breast cancer surgery (lumpectomy vs. mastectomy with or without axillary dissection), and chemotherapy regimens were recorded. Written consent to access medical records was obtained from all participants on the study.

### DIBH technique, treatment planning, and evaluation metrics

Per institutional policy, all patients with left-sided breast cancer underwent free-breathing (FB) and DIBH CT simulations and RT planning in the supine position on a breast board with arms raised above the head. DIBH was combined with infrared optical tracking for body surface imaging for accurate and reproducible patient set-up (AlignRT, VisionRT, London, United Kingdom) as previously described (14). Portal films and cone-beam CT scans were used as needed.

Target volumes and organs at risk (OARs) were contoured as previously described (15). Heart contours extended superiorly to the bifurcation of the pulmonary artery. Treatment plans for both the FB and DIBH scans were generated using Prowess® Panther 5.10 (Prowess, Chico, CA). Two opposed tangent fields were typically used for radiation delivery to the breast or chest wall with or without the “field-in-field” technique to further improve dose homogeneity as needed. Additional fields were used to treat the supraclavicular, axillary, or internal mammary lymph nodes, when needed. When 3 fields (2 tangents and a supraclavicular field) were needed, a single mono-isocentric technique with half-beam blocks was used.

Volume delineation was done according to RTOG 1304/NSABP-B51 protocol. Maximum acceptable point dose was ≤ 115% and ≥95% PTV received 95% of the prescribed dose of 50 Gy in 25 fractions. Mean heart dose (MHD) and volume of heart receiving 4 Gy (V4) were collected retrospectively using dose-volume histograms from both FB and DIBH plans. These parameters were selected as MHD (even at low doses) has been associated with increased cardiovascular risk and MHD < 4 Gy is commonly adopted in treatment planning sections of clinical trials (16). Timing of CT simulation was stratified into the four known seasons (winter, spring, summer, and fall). Field size was defined as the area being treated by radiation in cm^2^. Time surgery-CT was defined as the time in days from mastectomy or lumpectomy to CT simulation.

### Statistics

Continuous variables were reported as mean ± standard deviation. Fisher's exact test and Chi-square test were used for univariate analysis and the stepwise method was used for multi-variable analysis. Pearson correlation coefficient was used for correlational analysis. *T*-test and analysis of variance (ANOVA) were used for group comparisons as needed. The Statistical Package for Social Sciences (SPSS version 24.0) was used for all statistical analysis. A *p* ≤ 0.05 in two tailed tests was considered statistically significant for all comparisons.

## Results

### Patient characteristics and treatment planning

One-hundred and three patients were included in this study. Mean age was 51.9 ± 11.8 years. Most patients had T1-2 (88.2%) and node positive (64.1%) disease. Majority of patients did not have any relevant simultaneous comorbidities including lung diseases (98.9% without lung disease), congestive heart failure (98.9% without CHF), psychiatric illness (94% without psychiatric conditions), or prior chest surgery (96% without prior chest surgery). All patients had left-sided breast cancer and the majority underwent mastectomy (97%) and axillary dissection (62.7%). Baseline patient characteristics are reported in Table 1.

**Table 1:** Baseline patient and treatment characteristics.

**Characteristics**		***N* (%)**
Age (years)	Mean (± SD)	51.87 (± 11.83)
BMI (kg/m^2^)	Mean (± SD)	27.98 (± 5.17)
Smoking	Yes (%) No (%)	52 (59.8) 35 (40.2)
Tumor stage	T0 (%) T1 (%) T2 (%) T3 (%) T4 (%)	1 (1) 55 (53.9) 35 (34.3) 10 (9.8) 1 (1)
Nodal involvement	Yes (%) No (%)	66 (64.1) 37 (35.9)
Lung disease	Yes (%) No (%)	1 (1.1) 87 (98.9)
CHF	Yes (%) No (%)	1 (1.1) 87 (98.9)
Prior chest surgery	Yes (%) No (%)	4 (4) 96 (96)
Psychiatric disease	Yes (%) No (%)	4 (6) 63 (94)
Surgery type	Lumpectomy (%) Mastectomy (%)	3 (2.9) 99 (97.1)
Axillary dissection	YES (%) NO (%)	64 (62.7) 38 (37.3)
Chemo	Yes (%) No (%)	46 (45.1) 56 (54.9)
Chemo regimen	AC (%) AC/T (%) EC/T (%) FEC/T (%) Others (%)	8 (16.3) 25 (51) 7 (14.3) 4 (8.2) 5 (10)
Time Surgery-CT (days)	Mean (± SD)	108.14 (± 100.49)
Seasons (CT simulation)	Winter (%)	41 (39.8)
	Spring (%)	19 (18.4)
	Summer (%)	27 (26.2)
	Fall (%)	16 (15.5)
Field size (cm^2^)	Mean (± SD)	218.62 (± 39.83)

*CHF, Congestive Heart Failure; AC, Adriamycin/cyclophosphamide; AC/T, Adriamycin, Cyclophosphamide/Taxanes; EC/T, Epirubicin, Cyclophosphamide/Taxanes; FEC/T, Fluorouracil, Epirubicin, Cyclophosphamide/Taxanes; CT, Computed Tomography; SD, standard deviation*.

**Figure 1:**
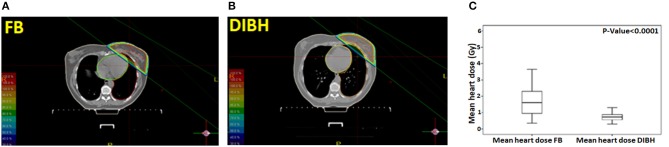
A representative treatment plan for a patient in both the free-breathing **(A)** and deep inspiration breath-hold **(B)** conditions. Box plot showing the mean heart dose (MHD) in free-breathing and deep inspiration breath-hold **(C)**.

All patients tolerated DIBH well. Representative treatment plans for a patient in both the FB and DIBH conditions are showed in Figures 1A,B showing the benefit with DIBH in physically removing the heart from beam trajectory. Mean heart dose (MHD) was significantly reduced in the DIBH plans (0.74 ± 0.25 Gy vs. 1.72 ± 0.98 Gy; *p* < 0.0001; Figure 1C). V4 was similarly decreased in the DIBH plans (4 ± 4.98 cc vs. 20.79 ± 18.2 cc; *p* < 0.0001).

### Predictors of cardiac sparing

**Figure 2:**
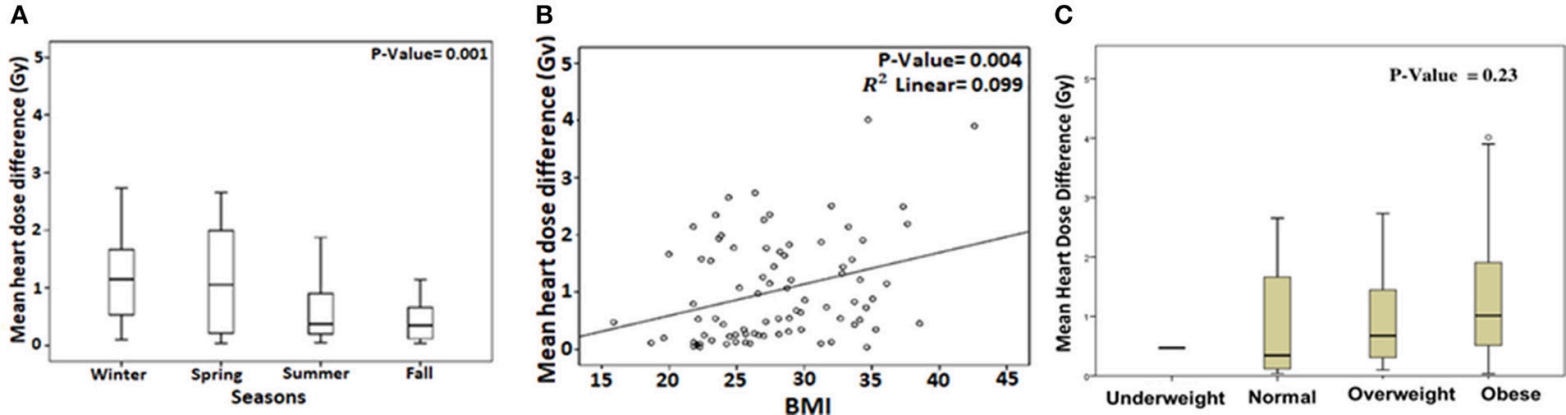
Box plot showing the mean heart dose difference (MHDD) between free-breathing and deep inspiration breath-hold with respect to timing of CT simulation **(A)**. Correlation between MHDD and body mass index (BMI) plotted as linear **(B)** and categorical variable **(C)**.

We analyzed predictors of cardiac sparing with DIBH using univariate analysis of all factors presented in Table 1. We used the difference in MHD and V4 between FB and DIBH scans as surrogates of cardiac sparing. Larger body mass index (BMI; *p* = 0.004), non-smoking status (*p* = 0.05), and spring/winter timing of CT simulation (*p* = 0.001) were associated with larger MHD difference between FB and DIBH plans (Table 2 and Figures 2A–C). A trend to higher MHDD was noted in obese patients when BMI was plotted as categorical variable (Figure 2C). Similarly, larger body mass index (BMI; *p* = 0.005), larger field size (*p* = 0.027), having undergone axillary dissection (*p* = 0.04), prior chemotherapy (*p* = 0.05), and spring/winter timing of CT simulation (*p* = 0.009) were associated with larger V4 difference (Table 3).

**Table 2:** Univariate analysis of different potential predictors of MHDD (mean heart dose difference) between free-breathing and breath-hold scans.

**Characteristics**		**rMHDD/MHDD (± SD)**	***P*-Value**
		**rMHDD**
Age (years)	–	−0.091	0.359
BMI (kg/m^2^)	–	0.314	0.004
Field size (cm^2^)	–	0.182	0.066
Time Surgery-CT (days)	–	0.021	0.837
		**MHDD (**± **SD)**
Smoking	No	1.19 ± 0.89	0.05
	Yes	0.82 ± 0.79
Tumor stage	T0	0.53	0.987
	T1	0.99 ± 0.95
	T2	0.99 ± 0.74
	T3	0.94 ± 0.93
	T4	0.81
Nodal involvement	No	1.06 ± 0.87	0.224
	Yes	0.84 ± 0.85
Lung disease	No	0.97 ± 0.89	0.599
	Yes	1.45
CHF	No	0.98 ± 0.89	0.993
	Yes	0.98
Prior chest surgery	No	0.98 ± 0.86	0.272
	Yes	1.47 ± 0.94
Psychiatric disease	No	1.04 ± 0.94	0.639
	Yes	0.81 ± 0.51
Surgery type	Lumpectomy	0.41 ± 0.39	0.25
	Mastectomy	0.99 ± 0.87
Axillary dissection	No	0.98 ± 0.87	0.972
	Yes	0.99 ± 0.87
Chemo	No	0.99 ± 0.91	0.97
	Yes	0.98 ± 0.83
Seasons (CT simulation)	Winter	1.27 ± 0.91
	Spring	1.22 ± 0.96	0.001
	Summer	0.66 ± 0.68
	Fall	0.47 ± 0.45

*MHDD, Mean heart dose difference between free-breathing and breath-hold scans; rMHDD, Correlation with MHDD; CHF, Cardiac heart failure; CT, Computed Tomography; SD, standard deviation*.

**Table 3:** Univariate analysis of different potential predictors of heart DV4 (difference in heart volume receiving 4 Gy or more) between free-breathing and breath-hold scans.

**Characteristics**		**Heart DV4**		***P*-Value**
		** ≤ 2.49 *N* = 52**	**>2.49 *N* = 51**
Age (years)	Mean (± SD)	53.79 ± 11.82	49.92 ± 11.61	0.097
BMI (kg/m^2^)	Mean (± SD)	26.40 ± 4.64	29.57 ± 5.25	0.005
Smoking	Yes	19 (46.3)	16 (34.8)	0.27
Tumor stage	T0	1 (2)	0 (0)	0.61
	T1	30 (58.8)	25 (49)
	T2	16 (31.4)	19 (37.3)
	T3	4 (7.8)	6 (11.8)
	T4	0 (0)	1 (2)
Nodal involvement	Yes	19 (36.5)	18 (35.3)	0.89
Lung disease	Yes	0 (0)	1 (1)	0.46
CHF	Yes	1 (2.1)	0 (0)	1
Prior chest surgery	Yes	2 (4.1)	2 (3.9)	1
Psychiatric disease	Yes	2 (5.9)	2 (6.1)	1
Surgery type	Lumpectomy	2 (3.8)	1 (2)	1
	Mastectomy	50 (96.2)	49 (98)
Axillary dissection	Yes	14 (27.5)	24 (47.1)	0.04
Chemo	Yes	23 (45.1)	33 (64.7)	0.05
Time Surgery-CT (days)	Mean (± SD)	91.48 ± 79.60	124.14 ± 115.69	0.108
Seasons (CT simulation)	Winter	14 (26.9)	27 (52.9)
	Spring	8 (15.4)	11 (21.6)	0.009
	Summer	18 (34.6)	9 (17.6)
	Fall	12 (23.1)	4 (7.8)
Field size (cm^2^)	Mean (± SD)	210.09 ± 40.60	227.31 ± 37.44	0.027

*Heart DV4, Difference in heart volume having 4Gy or more between free-breathing and breath-hold scans (normalized to initial heart volume); CHF, Cardiac heart failure; BMI, body mass index; CT, Computed Tomography; SD, standard deviation*.

A multi-variate model was performed for the variables which were significant on univariate analysis. Only BMI (*p* = 0.001) and timing of CT simulation (*p* = 0.001) continued to predict larger MHD difference between FB and DIBH plans (Table 4). Similarly, only BMI (*p* = 0.03) and timing of CT simulation (*p* = 0.003 and *p* = 0.01) continued to predict larger V4 difference (Table 4).

**Table 4:** Multivariate analysis for the predictors of MHDD (mean heart dose difference) or heart DV4 (difference in heart volume receiving 4 Gy or more).

**Variables**	**β (95 % CI)**	***P*-value**
**MHDD**^a^		
BMI	0.05 (0.02; 0.09)	0.001
Season-Summer	−0.76 (−1.19; −0.34)	0.001
Season-Fall	−0.60 (−0.95; −0.25)	0.001
**Variables**	**OR (95 % CI)**	***P*****-value**
**Heart DV4 (reference:**<**2.49)**^b^		
BMI	1.13 (1.01–1.27)	0.03
Season-Summer	0.20 (0.07–0.59)	0.003
Season-Fall	0.14 (0.03–0.57)	0.01

a *Variables included in the model were BMI, field size, smoking (reference: no), season (reference: winter)*.

b *Variables included in the model were BMI, field size, axillary dissection, chemo, season (reference: winter)*.

*BMI, body mass index; OR, odds ratio; MHDD, difference in mean heart dose between free-breathing and breath-hold scans; Heart DV4, difference in heart volume receiving 4 Gy or more between free-breathing and breath-hold scans*.

## Discussion

In this study, we evaluated non-dosimetric predictors of cardiac sparing with the use of deep inspiration breath-hold (DIBH) in patients with left breast cancer undergoing RT, majority of which were to the chest wall after mastectomy. We identified BMI and timing of CT simulation as significant predictors of benefit from DIBH scans in terms of reduction in MHD or V4. These results are mostly applicable to patients after mastectomy given that most of our patients (97%) underwent mastectomy.

Treatment of left breast cancer benefits from respiratory gating using surface motion tracking systems. This study was carried out in a tertiary care center in a middle income country and was motivated by the need to conserve resources used in treating patients requiring complex motion management systems which not only require an initial capital investment in such systems but also in personnel training and may lead to increased treatment times. We have used the system for 10 years and similar to others, we have confirmed that the implementation of DIBH is feasible, efficacious in reducing cardiac exposure and, adds few extra minutes to treatment time (17).

Intuitively, patients with unfavorable cardiac anatomy where the heart extends into the treatment fields benefit the most from DIBH. Similarly, patients are more likely to benefit from DIBH after mastectomy compared to lumpectomy (18, 19). Recent studies investigated several candidate predictors of cardiac benefit from DIBH with many potential dosimetric measures such as maximal heart distance, cardiac contact distance, and heart V_50%_>10 cm^3^ (volume >10 cm^3^ of the heart receiving 50% of the prescribed dose) (10–13). While these measures allowed a better understanding of benefit after DIBH and a better selection of patients, our study sought to identify patient-related non-dosimetric factors that may aid the decision to select patients for DIBH even before the breath hold scan is performed.

Several factors were identified in our study but only BMI and timing of CT simulation were statistically significant on multi-variate analysis. Larger BMI was associated with increased MHD difference after DIBH. Previously, the relevance of BMI to benefit after DIBH has been controversial. One study using active breathing control (ABC) DIBH showed that BMI did not correlate with cardiac sparing (20) in contrary to other studies which showed the opposite (21, 22). In fact, the correlation between BMI and change in MHD corresponds to to 6.06 cGy/kg/m^2^ (22).It has been proposed by others that heart motion could differ with increased body habitus (22). Most interestingly, patients who underwent treatment planning in winter/spring reaped more cardiac sparing than those in summer/fall. This is possibly related to the seasonal variations in lung function with a predicted decline in lung function from July to September corresponding to the summer/fall seasons (23). Alternatively, this could be due to the high humidity in Beirut during summer time.

The current study is limited by its retrospective nature and the relatively low mean heart dose in our cohort in the FB conditions indicating that the majority of patients had favorable cardiac anatomy. This, however, probably reflects a real life breast cancer patient cohort in a tertiary care center in the Middle East. Indeed, DIBH benefit is not limited to patients with unfavorable cardiac anatomy as shown previously (13). While the question of conserving resources is extremely important in low and middle income countries, it is equally important in high income countries as well. The large number of cases in this study reflecting a representative population of patients with breast cancer increases its significance in all radiotherapy centers. Another limitation of this study is the absence of detailed dosimetric analysis of sub-cardiac structures whose exposure has been linked to deleterious cardiac events such as the left anterior descending artery (24, 25). The absence of specific dose constraints to these structures precluded such detailed analysis. In addition, the clinical relevance of heart V4 needs to be validated in prospective studies. Finally, the correlation between DIBH benefit and CT simulation time is limited to cities around the Mediterranean with seasonal weather like that in Beirut. Radiation oncologists are thus urged to utilize all possible techniques to reduce cardiac dose in patients undergoing RT for left sided breast cancer. Whereas, the long-term consequences of cardiac exposure during left breast/chest wall RT remain controversial at best, RT causes volume-dependent perfusion defects within 2 years of RT with corresponding wall-motion abnormalities (26). Surface motion tracking is a very powerful tool to this effect but it may not be feasible to apply for all patients in the setting of limited resources due to additional cost, time and labor associated with its implementation. In addition to dosimetric parameters such as maximum heart distance and cardiac contact distance, BMI and timing of treatments can be used to aid in patient selection for DIBH treatment. Further work is needed to eventually be able to create an algorithm which will identify those patients who are ideal candidates for DIBH scans. In conclusion, whenever resources are restrained our data suggests that patients BMI needs to be taken into account when considering DIBH treatment technique.

## Author contributions

AM conceptualization, data curation, formal analysis, investigation, methodology, writing, and editing. OM data curation, formal analysis, writing, and editing. PR data curation, formal analysis, investigation. RT, MM, and HT: data curation, formal analysis, investigation, methodology. WJ, BY, TE, FG, and BS conceptualization, data curation, formal analysis, investigation, methodology. YZ conceptualization, data curation, formal analysis, investigation, methodology, supervision, writing—original draft, and writing—review.

### Conflict of interest statement

The authors declare that the research was conducted in the absence of any commercial or financial relationships that could be construed as a potential conflict of interest.

## References

[1] EBCTCG (Early Breast Cancer Trialists' Collaborative Group) McGale P Taylor C Correa C Cutter D Duane F Effect of radiotherapy after mastectomy and axillary surgery on 10-year recurrence and 20-year breast cancer mortality: meta-analysis of individual patient data for 8135 women in 22 randomised trials. Lancet (2014) 383:2127–35. 10.1016/S0140-6736(14)60488-8 24656685 PMC5015598

[2] Clarke M Collins R Darby S Davies C Elphinstone P Evans V. Effects of radiotherapy and of differences in the extent of surgery for early breast cancer on local recurrence and 15-year survival: an overview of the randomised trials. Lancet (2005) 366:2087–106. 10.1016/S0140-6736(05)67887-7 16360786

[3] Early Breast Cancer Trialists' Collaborative Group (EBCTCG) Darby S McGale P Correa C Taylor C Arriagada R Effect of radiotherapy after breast-conserving surgery on 10-year recurrence and 15-year breast cancer death: meta-analysis of individual patient data for 10,801 women in 17 randomised trials. Lancet (2011) 378:1707–16. 10.1016/S0140-6736(11)61629-2 22019144 PMC3254252

[4] Wadsten C Wennstig AK Garmo H Nilsson G Blomqvist C Holmberg L. Risk of ischemic heart disease after radiotherapy for ductal carcinoma *in situ*. Breast Cancer Res Treat. (2018) 171:95-101. 10.1007/s10549-018-4803-1 29730730

[5] Højris I Overgaard M Christensen JJ Overgaard J Morbidity and mortality of ischaemic heart disease in high-risk breast-cancer patients after adjuvant postmastectomy systemic treatment with or without radiotherapy: analysis of DBCG 82b and 82c randomised trials. Radiotherapy Committee of the Danish Breast Cancer Cooperative Group. Lancet (1999) 354:1425–30. 10.1016/S0140-6736(99)02245-X 10543669

[6] Darby SC McGale P Taylor CW Peto R. Long-term mortality from heart disease and lung cancer after radiotherapy for early breast cancer: prospective cohort study of about 300,000 women in US SEER cancer registries. Lancet Oncol. (2005) 6:557–65. 10.1016/S1470-2045(05)70251-5 16054566

[7] Taylor CW Wang Z Macaulay E Jagsi R Duane F Darby SC. Exposure of the heart in breast cancer radiation therapy: a systematic review of heart doses published during 2003 to 2013. Int J Radiat Oncol Biol Phys. (2015) 93:845–53. 10.1016/j.ijrobp.2015.07.2292 26530753

[8] Nissen HD Appelt AL. Improved heart, lung and target dose with deep inspiration breath hold in a large clinical series of breast cancer patients. Radiother Oncol. (2013) 106:28–32. 10.1016/j.radonc.2012.10.016 23199652

[9] Swanson T Grills IS Ye H Entwistle A Teahan M Letts N Six-year experience routinely using moderate deep inspiration breath-hold for the reduction of cardiac dose in left-sided breast irradiation for patients with early-stage or locally advanced breast cancer. Am J Clin Oncol. (2013) 36:24–30. 10.1097/COC.0b013e31823fe481 22270108 PMC3375337

[10] Kong FM Klein EE Bradley JD Mansur DB Taylor ME Perez CA. The impact of central lung distance, maximal heart distance, and radiation technique on the volumetric dose of the lung and heart for intact breast radiation. Int J Radiat Oncol Biol Phys. (2002) 54:963–71. 10.1016/S0360-3016(02)03741-0 12377351

[11] Rochet N Drake JI Harrington K Wolfgang JA Napolitano B Sadek BT3. Deep inspiration breath-hold technique in left-sided breast cancer radiation therapy: evaluating cardiac contact distance as a predictor of cardiac exposure for patient selection. Pract Radiat Oncol. (2015) 5:e127–34. 10.1016/j.prro.2014.08.003 25413399

[12] Wang W Purdie TG Rahman M Marshall A Liu FF Fyles A. Rapid automated treatment planning process to select breast cancer patients for active breathing control to achieve cardiac dose reduction. Int J Radiat Oncol Biol Phys. (2012) 82:386–93. 10.1016/j.ijrobp.2010.09.026 21093165

[13] Mohamad O Shiao J Zhao B Roach K Ramirez E Vo DT. Deep inspiration breathhold for left-sided breast cancer patients with unfavorable cardiac anatomy requiring internal mammary nodal irradiation. Pract Radiat Oncol. (2017) 7:e361–7. 10.1016/j.prro.2017.04.006 28666899

[14] Gierga DP Turcotte JC Sharp GC Sedlacek DE Cotter CR Taghian AG. A voluntary breath-hold treatment technique for the left breast with unfavorable cardiac anatomy using surface imaging. Int J Radiat Oncol Biol Phys. (2012) 84:e663–8. 10.1016/j.ijrobp.2012.07.2379 22975605

[15] Gentile MS Usman AA Neuschler EI Sathiaseelan V Hayes JP Small W Jr. Contouring guidelines for the axillary lymph nodes for the delivery of radiation therapy in breast cancer: evaluation of the RTOG breast cancer atlas. Int J Radiat Oncol Biol Phys (2015) 93:257–65. 10.1016/j.ijrobp.2015.07.002 26383674 PMC12422023

[16] Darby SC Ewertz M McGale P Bennet AM Blom-Goldman U Brønnum D. Risk of ischemic heart disease in women after radiotherapy for breast cancer. N. Engl. (2013) 368:987–98. 10.1056/NEJMoa1209825 23484825

[17] Comsa D Barnett E Le K Mohamoud G Zaremski D Fenkell L. Introduction of moderate deep inspiration breath hold for radiation therapy of left breast: initial experience of a regional cancer center. Pract Radiat Oncol. (2014) 4:298–305. 10.1016/j.prro.2013.10.006 25194098

[18] Lin A Sharieff W Juhasz J Whelan T Kim DH. The benefit of deep inspiration breath hold: evaluating cardiac radiation exposure in patients after mastectomy and after breast-conserving surgery. Breast Cancer (2017) 24:86–91. 10.1007/s12282-016-0676-5 26886584

[19] Yeung R Conroy L Long K Walrath D Li H Smith W. Cardiac dose reduction with deep inspiration breath hold for left-sided breast cancer radiotherapy patients with and without regional nodal irradiation. Radiat Oncol. (2015) 10:200. 10.1186/s13014-015-0511-8 26391237 PMC4578779

[20] Barry A Rock K Sole C Rahman M Pintilie M Lee G. The impact of active breathing control on internal mammary lymph node coverage and normal tissue exposure in breast cancer patients planned for left-sided postmastectomy radiation therapy. Pract Radiat Oncol. (2017)7:228–33. 10.1016/j.prro.2016.11.010 28139424

[21] Czeremszynska B Drozda S Górzynski M Kepka L. Selection of patients with left breast cancer for deep-inspiration breath-hold radiotherapy technique: results of a prospective study. Rep Pract Oncol Radiother. (2017) 22:341–8. 10.1016/j.rpor.2017.05.002 28701900 PMC5496478

[22] Tanguturi SK Lyatskaya Y Chen Y Catalano PJ Chen MH Yeo WP. Prospective assessment of deep inspiration breath-hold using 3-dimensional surface tracking for irradiation of left-sided breast cancer. Pract Radiat Oncol. (2015) 5:358–65. 10.1016/j.prro.2015.06.002 26231594

[23] Strachan P Medarov BI Seasonal variations in lung function. Chest (2005) 128:173S. 10.1378/chest.128.4MeetingAbstracts.173S-a/

[24] Nilsson G Holmberg L Garmo H Duvernoy O Sjögren I Lagerqvist B. Distribution of coronary artery stenosis after radiation for breast cancer. J Clin Oncol. (2012) 30:380–6. 10.1200/JCO.2011.34.5900 22203772

[25] Sardaro A Petruzzelli MF D'Errico MP Grimaldi L Pili G Portaluri M. Radiation-induced cardiac damage in early left breast cancer patients: risk factors, biological mechanisms, radiobiology, and dosimetric constraints. Radiother Oncol. (2012) 103:133–42. 10.1016/j.radonc.2012.02.008 22391054

[26] Marks LB Yu X Prosnitz RG Zhou SM Hardenbergh PH Blazing M. The incidence and functional consequences of RT-associated cardiac perfusion defects. Int J Radiat Oncol Biol Phys. (2005) 63:214–23. 10.1016/j.ijrobp.2005.01.029 16111592

